# Machine Learning-based Derivation and External Validation of a Tool to Predict Death and Development of Organ Failure in Hospitalized Patients with COVID-19

**DOI:** 10.21203/rs.3.rs-1009310/v1

**Published:** 2021-11-16

**Authors:** Yixi Xu, Anusua Trivedi, Nicholas Becker, Marian Blazes, Juan Ferres, Aaron Lee, W. Liles, Pavan Bhatraju

**Affiliations:** Microsoft (United States); Microsoft (United States); Microsoft (United States); University of Washington; Microsoft (United States); University of Washington; University of Washington; University of Washington

**Keywords:** COVID-19, hospitalized patients, prediction, mortality, organ failure

## Abstract

**Background:**

COVID-19 mortality risk stratification tools could improve care, inform accurate and rapid triage decisions, and guide family discussions regarding goals of care. A minority of COVID-19 prognostic tools have been tested in external cohorts. Our objective was to compare machine learning algorithms and develop a tool for predicting subsequent clinical outcomes in COVID-19.

**Methods:**

We conducted a retrospective cohort study that included hospitalized patients with COVID-19 from March 2020 to March 2021. 712 consecutive patients from University of Washington (UW) and 345 patients from Tongji Hospital in China were included. We applied three different machine learning algorithms to clinical and laboratory data collected within the initial 24 hours of hospital admission to determine the risk of in-hospital mortality, transfer to the intensive care unit (ICU), shock requiring vasopressors, and receipt of renal replacement therapy (RRT). Mortality risk models were derived, internally validated in UW and externally validated in Tongji Hospital. The risk models for ICU transfer, shock and RRT were derived and internally validated in the UW dataset.

**Results:**

Among the UW dataset, 122 patients died (17%) during hospitalization and the mean days to hospital mortality was 15.7 +/− 21.5 (mean +/− SD). Elastic net logistic regression resulted in a C-statistic for in-hospital mortality of 0.72 (95% CI, 0.64 to 0.81) in the internal validation and 0.85 (95% CI, 0.81 to 0.89) in the external validation set. Age, platelet count, and white blood cell count were the most important predictors of mortality. In the sub-group of patients > 50 years of age, the mortality prediction model continued to perform with a C-statistic of 0.82 (95% CI:0.76,0.87). Mortality prediction models also performed well for shock and RRT in the UW dataset but functioned with lower accuracy for ICU transfer.

**Conclusions:**

We trained, internally and externally validated a prediction model using data collected within 24 hours of hospital admission to predict in-hospital mortality on average two weeks prior to death. We also developed models to predict RRT and shock with high accuracy. These models could be used to improve triage decisions, resource allocation, and support clinical trial enrichment.

## Introduction

The ongoing COVID-19 pandemic, caused by human infection with SARS-CoV-2, has been a major cause of mortality worldwide[1]. A robust public health and biomedical response to a pandemic is contingent on timely and accurate information, including rapid diagnosis and assessment of patients at risk for severe disease[2]. A clinical model, incorporating recognized risk factors and clinical features, that could effectively identify individuals at risk for severe disease and adverse clinical outcomes could greatly assist with rational triage and resource allocation[3].

Sequential Organ Failure Assessment (SOFA) score has been widely used to assist with triage of patients with COVID-19. However, the accuracy of SOFA for predicting mortality in COVID-19 is poor (AUC of 0.59 (95% CI, 0.55 – 0.63), possibly because SOFA was developed in patients with various and alternative forms of sepsis[4]. While multiple papers have focused on the development of prognostic models to predict mortality risk using demographic and clinical data, these papers have had limited validation in external patient cohorts[5-9]. For example, one prediction model that used three blood biomarkers initially reported a 90% accuracy to predict mortality. However, when this model was tested in an external cohort, reported accuracy declined to only 40-50%[10, 11]. Previous COVID-19 prediction models have been limited in reporting how features were selected, timing of variable collection and outcomes and calibration performance of the model[5, 6].

To date, COVID-19 prediction models have largely focused on mortality[5, 12, 13], rather than risk for specific organ dysfunction, such as hypotension requiring vasopressors (shock), renal failure requiring renal replacement therapy (RRT), or hypoxemic respiratory failure requiring invasive mechanical ventilation. An accurate means to predict risk for specific organ injury in severe COVID-19 would greatly assist clinical decision-making. Studies have attempted to assess such risks by grouping several outcomes of interest together and building a predictive model[13-16]. Despite the success of this kind of model, grouping the outcomes together is less useful for resource allocation and triage, as patients will require different equipment and staffing expertise depending on their disease course and complications[3, 17]. To address this concern, we created separate models to predict risk of in-hospital mortality, ICU transfer, shock, and renal replacement therapy (RRT) based on demographic and clinical information collected on the first day of hospital admission. We then used an open source COVID-19 dataset to validate our mortality prediction model. Additional outcomes, such as ICU transfer, shock and need for RRT, were not available in the external validation set.

## Methods

### Study design and patient population

The University of Washington (UW) dataset includes demographic and clinical data from COVID-19 positive patients who were admitted to *two hospitals at the UW (Montlake and Harborview campuses)* between March, 2020 and March, 2021.

The COVID-19 dataset at Tongji Hospital dataset has been previously reported[6]. In brief, patients from the Tongji COVID-19 dataset were enrolled from January 10^th^ to February 18^th^, 2020. Patients from the Tongji dataset made the external validation cohort for the mortality model. In the UW and Tongji datasets, mortality prediction models were developed using clinical data collected on the first day of hospital admission.

### Ethics approval and consent to participate

The University of Washington institutional review board (IRB) approved the study protocol (STUDY10159). All clinical investigations were conducted based on the principles expressed in the declaration of Helsinki. Written informed consent was waived by the IRB due to the retrospective nature of our study of routine clinical data.

### Outcomes

The primary outcome was in-hospital mortality. We developed and internally validated a prediction model for in-hospital mortality and externally validated the model in the Tongji dataset. Secondary outcomes were ICU transfer, shock and receipt of RRT. These secondary outcomes were missing in the Tongji dataset and so we developed and cross-validated prediction models for secondary outcomes using the UW dataset.

### Feature Selection

The following steps were taken for feature selection. First, features were dropped if >10% of the values were missing. Second, near-zero variance features were removed, as these features almost exclusively had one unique value. Third, pair-wise correlations between all the features were calculated. If two features had a correlation larger than 0.8, the feature with a larger mean absolute correlation was dropped. Fourth, missing values were replaced by the mode if the variable was categorical or by the median otherwise. Finally, all the continuous variables were standardized.

Twenty features overlapped between the UW and Tongji Hospital datasets, and these 20 features were used for the mortality prediction model. All clinical and laboratory data were abstracted from the medical record within the first day of hospital admission, and patients were included in the analysis for each outcome only if the patients did not have the outcome on the first day of hospitalization. An individual prediction model was developed for each of the outcomes.

### Data partitioning, UW dataset

We randomly split the UW dataset into development and internal validation sets by stratified sampling. The training set included 475 patients, and the internal validation set included 237 patients. First, we trained models on the training set, and then selected the best model by its performance on the internal validation set. Top models for in-hospital mortality were then tested in the external validation set. We performed cross validation in the internal validation set for the three prediction models for ICU transfer, shock and RRT. We used the UW dataset as follows (1) patients were randomly split into 10 folds in a stratified fashion using the outcome variable; (2) the model was trained using nine of the ten folds and validated on the remaining fold. The procedure was repeated ten times until each fold had been used as a validation fold exactly once; (3). Steps 1 and 2 were repeated ten times.

### Machine learning models

*Least absolute shrinkage and selection operator (LASSO) logistic regression* is a logistic regression approach with L1 penalties[18]. The L1 penalty terms encourage sparsity, thus preventing overfitting and yielding a small model. A weighted LASSO logistic regression was used to handle the imbalanced data. The hyperparameter lambda was selected by stratified 10-fold cross validation.

*Elastic net logistic regression (LR)* is an approach that combines (LASSO LR and ridge logistic regression, incorporating both L1 and L2 penalties[19]. It can generate sparse models which outperform LASSO logistic regression when highly correlated predictors are present. The hyperparameters alpha and lambda were selected by stratified 10-fold cross validation.

*eXtreme Gradient Boosting* (XGBoost). XGBoost is a gradient boosted machine (GBM) based on decision trees that separate patients with and without the outcome of interest using simple yes-no splits, which can be visualized in the form of decision trees[20]. GBM builds sequential trees, such that each tree attempts to improve model fit by more highly weighting the difficult-to-predict patients. The following hyperparameter settings were applied: nrounds =150, eta = 0.2, colsample_bytree = .9, gamma = 1, subsample=.9 and max_depth=4.

#### Class imbalance handling

A weighted version of each of the three above methods was used to handle imbalanced data. For example, if there were 90 positives and 10 negatives, then a weight of 10 over 90 was assigned to a positive sample and a weight of one was assigned to a negative sample.

#### Probability calibration

Isotonic regression was used to calibrate the probabilities outputted by the machine learning models[21]. The calibration model was fitted on the training samples only.

### Model comparison

We tested the three machine learning methods (LASSO LR, elastic net LR, and XGBoost) independently to predict each outcome. Model performance was compared using the area under the receiver operatory characteristic curve (AUC) and 95% CI[22,23]. Top performing models for in-hosptial mortality in the internal validation cohort were then carried forward to the external validation cohort. Two-sided p-values <0.05 were considered statistically significant. All models were developed using R.

## Results

### Patient characteristics

A total of 1,057 patients were included in the analysis, 712 from UW and 345 from Tongji Hospital. Baseline characteristics for patients who died vs survived are shown in Table 1. In the UW cohorts, patients who died were older (median [IQR] age 66 [54-75] vs. 55 [41 -66] years), more likely to be male (70% vs. 61 %), had lower platelet count (median [IQR] 155 [114 -234] vs. 200 [155 – 265]), and higher white blood cell counts (median [IQR] 9.85 [7.01 – 14.44] vs. 7.87 [5.64 – 11.37]. In the Tongji cohort there was a similar difference in baseline characteristics between patients who died and survived during hospitalization.

### Machine learning model for in-hospital mortality

**Figure 1:**
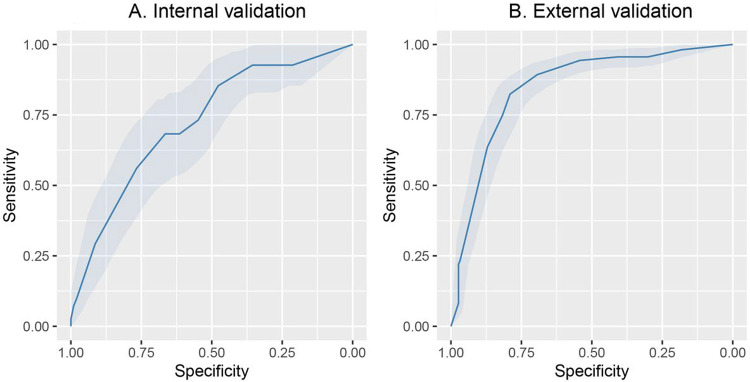
Receiver operator characteristics curves for mortality prediction. A. The c-statistic for in-hospital mortality using Elastic net LR model had an AUC of 0.72, 95% CI: 0.64 to 0.81 in the internal validation cohort. B. In the external validation cohort the model had an AUC of 0.85 (95% CI: 0.8 to 0.89) for in-hospital mortality.

**Figure 2:**
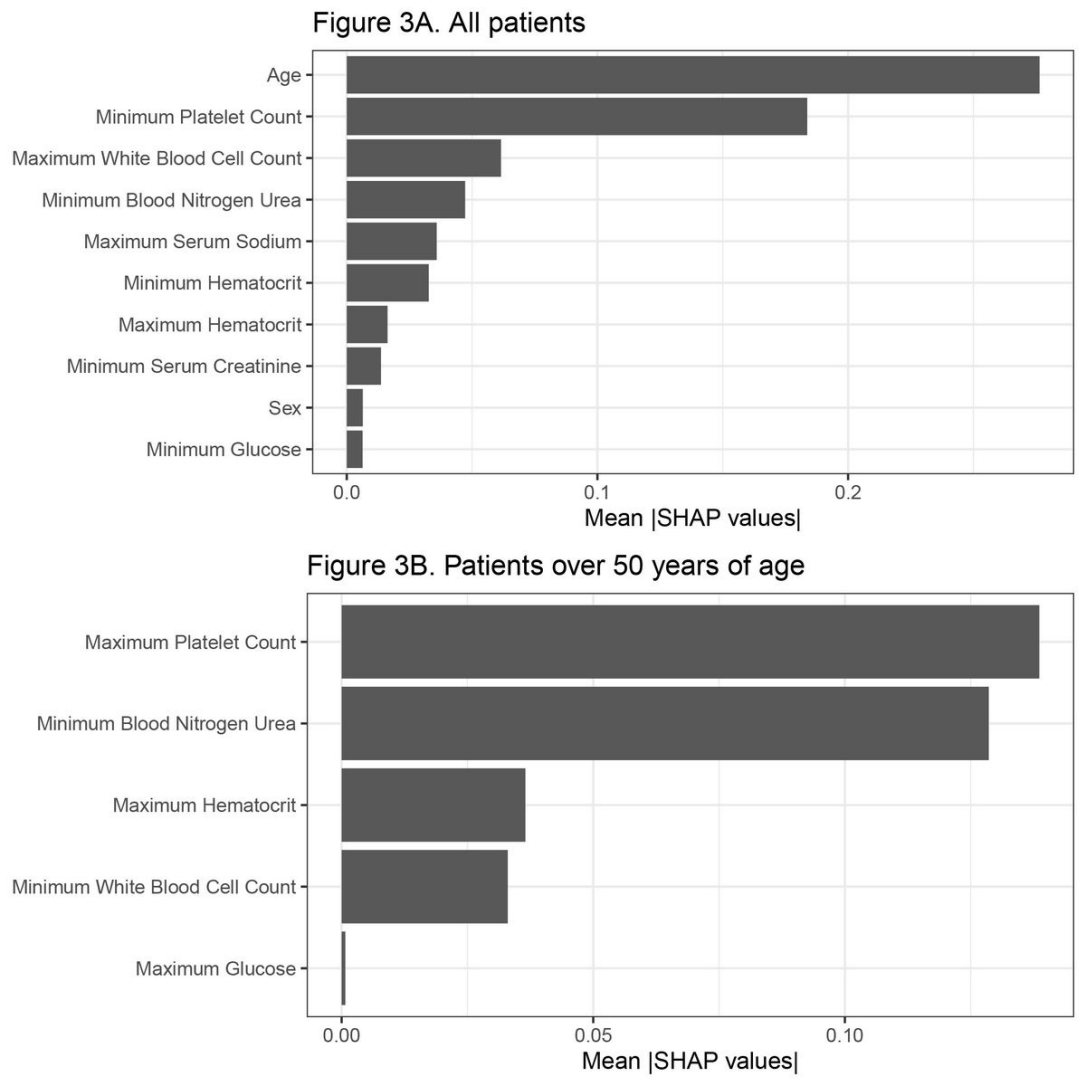
Variable importance plots for mortality in all patients and in patients over 50 years of age. A. Mean SHAP values are provided on the x-axis, which shows that age, minimum platelet count, maximum white blood cell count, minimum blood urea nitrogen, maximum serum sodium, minimum haematocrit, maximum hematocrit, minimum serum creatinine, sex and minimum glucose are the top-10 variables. B. Mean SHAP values are provided on the x-axis for the mortality prediction model in patients over 50 years of age, which includes the five selected variables: maximum platelet count, minimum blood urea nitrogen, maximum hematocrit, minimum white blood cell count, and maximum glucose.

Among 712 patients in the UW dataset, 122 (17%) died. The mean length of hospital stay was 15.7 (standard deviation 21.5) days for all patients and 14.8 (standard deviation 13.7) days for those that died. Among 328 patients from the Tongji Hospital dataset, 159 (46%) died. [24]. We applied three machine learning methods (LASSO LR, elastic net LR and XGBoost) to the training set and evaluated the model performance in the interval validation set. Elastic net LR model had the highest AUC in the internal validation set (0.72, 95% CI: 0.64 to 0.81) for in-hospital mortality. Next, we tested the elastic net LR model in the external validation cohort, and obtained an AUC of 0.85 (95% CI: 0.81 to 0.89) for in-hospital mortality (Figure 1A and 1B and Table 2A). The top 3 variables in the in-hospital mortality prediction model included, age, minimum platelet count, and maximum white blood cell count (Figure 2A).

**Figure 3:**
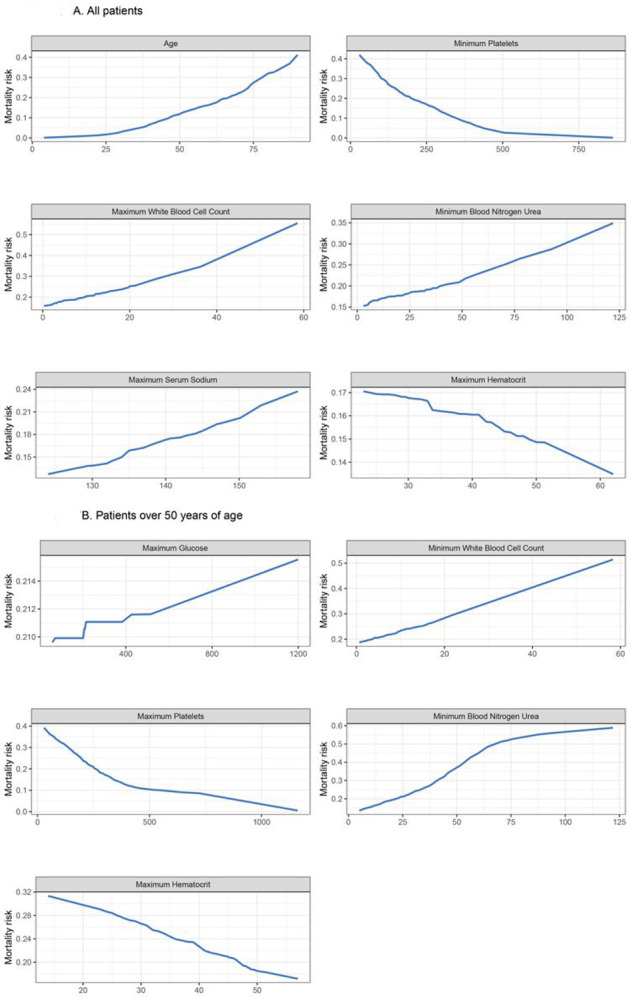
Partial dependence plots for mortality prediction model illustrating the relationship between mortality and the six top predictor variables A. Risk of mortality increases with increasing age, platelets < 500 109/L, and increasing white blood cell count. Risk of mortality increases with increasing blood urea nitrogen with an inflection point at 50 mg/dL. The risk of mortality increases with decreasing haematocrit levels and increasing sodium levels. B. Risk of mortality increases with increasing age, platelets < 500 109/L, and increasing white blood cell count. Risk of mortality increases with increasing blood urea nitrogen until 75 mg/dL and then levels off. The risk of mortality increases with decreasing haematocrit levels

Partial dependence plots for the most important continuous variables in elastic net LR are shown in Figure 3A. Older age was associated with a linear increase in mortality. In contrast, platelet count showed a relatively flat risk profile up to 500 x 10^9^/L after which risk of death increased linearly with lower platelet counts. The predicted risk of in-hospital mortality compared with the observed risk was well calibrated in the test set (Supplement Figure 1). In Table 3A, we provide the sensitivity, specificity, positive predictive values (PPV) and negative predictive values (NPV) across the three different cohorts for in-hospital mortality. We found that the model thresholds can be personalized to either maximize PPV or NPV. We found in the external validation cohort that the in-hospital mortality models had a maximum PPV and NPV of 0.84 or higher. Model coefficients are provided in Table S1 for future validation in diverse patient cohorts.

**Figure 4:**
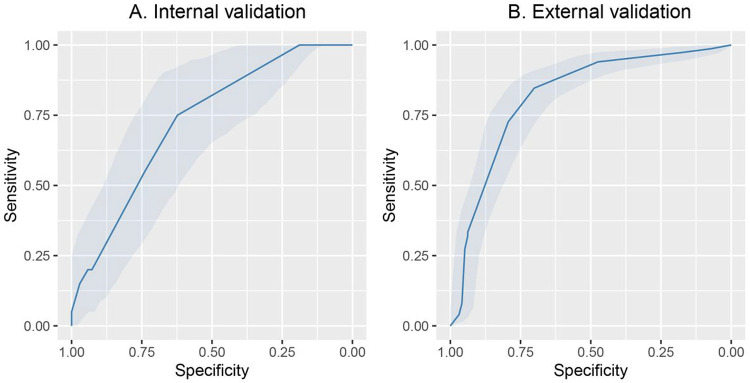
Receiver operator characteristics curves for ICU transfer, shock, RRT A. Receiver operator characteristics for ICU transfer in the cross-validation cohort. B. Receiver operator characteristics for shock in the cross-validation cohort C. Receiver operator characteristics for RRT in the cross-validation cohort.

To better understand the association between clinical features and in-hospital mortality, we concentrated on patients > 50 years of age and re-trained the models excluding age. Elastic net LR model had the highest AUC in the internal validation set (0.73, 95% CI: 0.61 to 84) for in-hospital mortality. Next, we tested the elastic net LR model in the external validation cohort and obtained an AUC of 0.82 (95% CI:0.76,0.87) for in-hospital mortality (Figure 4A and 4B and Table 2B). In Table 3B, we provide the sensitivity, specificity, positive predictive values (PPV) and negative predictive values (NPV) across the three different cohorts for in-hospital mortality in patients > 50 years of age. Partial dependence plots for the most important continuous variables in elastic net LR are shown in Figure 3B. Platelet count, blood nitrogen urea, haematocrit and white blood cell count were the top 4 variables that predicted in-hospital mortality in the patients > 50 years of age.

### Machine learning models for secondary outcomes

**Figure 5:**
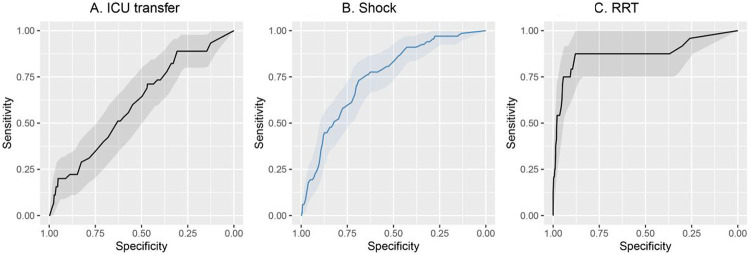
Receiver operator characteristics curves for mortality prediction for patients over 50. A. The c-statistic for in-hospital mortality using Elastic net LR in patients older than 50 had an AUC of 0.73, 95% CI: 0.61 to 0.84 in the internal validation cohort. B. In the external validation cohort the model had an AUC of 0.82 (95% CI: 0.7 to ,0.87)

We next developed and cross-validated prediction models for ICU transfer, shock and receipt of RRT. For the outcome of ICU transfer, 419 patients from the UW dataset were included in the training set, with 45 (11%) patients being transferred to the ICU within 28 days of admission. Patients were excluded from this analysis who were transferred to the ICU within the first day of hospitalization. The mean length of time to be transferred to ICU was 7.6 (standard deviation 9.1) days. Lasso LR achieved the highest AUC (0.60, 95% CI: 0.52,0.68) for prediction of ICU transfer compared with the other two methods (elastic net LR, XGBoost) (Figure 5A and Table 4). The two predictors that most strongly correlated with subsequent ICU transfer were age and minimum SpO_2_.

For the outcome of shock, 606 patients from the *UW* dataset were included in the training set, with 67 patients developing shock within 28 days of admission. Patients were excluded from this analysis who had shock within the first day of hospitalization. The mean length of time to develop shock was 7.0 +/− 6.5 days (mean +/− SD). Elastic net LR achieved the highest AUC of the three methods (0.76, 95% CI: 0.69 to 0.82) (Figure 5B and Table 4). The three predictors that were most highly correlated with subsequent development of shock were ICU admission, minimum mean arterial blood pressure and minimum Glasgow coma scale score.

For the outcome of receipt of RRT, 671 patients from the UW dataset were included in the training set with 24 patients receiving RRT within 28 days of admission. Patients were excluded from this analysis who received RRT within the first day of hospitalization. The mean length of time to receive RRT was 5.8 +/− 7.2 days (mean +/− SD). As shown in Figure 5C and Table 4, Lasso LR achieved a slightly higher mean AUC compared with the other two methods (0.88, 95% CI: 0.79 to 0.98). The predictor that most strongly influenced need for RRT was minimum serum creatinine. Variable importance plots for all the secondary outcomes can be found in Figure S2. Model calibration plots for each of the secondary outcomes are provided in Figure S1. Coefficients for variables are provided in Tables S2-S4.

## Discussion

In this derivation, internal validation and external validation study of adult hospitalized patients with COVID-19, we developed and validated an in-hospital mortality prediction tool using variables that are routinely collected within 24 hours of hospital admission. We found the mortality prediction model had high accuracy to predict mortality with a 2-week lead-time. We also found that elastic net logistic regression had the highest performance and best calibration of the machine learning models tested. In addition, we derived models for ICU transfer, shock and RRT. Our results provide a simple bedside tool and highlight clinical variables that can inform triage decisions and clinical care in hospitalized patients with COVID-19.

The mortality prediction tool was derived using 20 variables and exported to an external dataset. The model had higher discrimination in the external dataset, demonstrating the generalizability of the model. Variables that informed model development included age, white blood count, and platelet count. These variables have been individually shown to be previously prognostic in COVID-19 hospitalization as well as in sepsis[25-27]. A machine learning study in Germany for mortality prediction in COVID-19, also found that age and markers of thrombotic activity were predictive of ICU survival[28]. An advantage of our model to other studies is that we included not only patients admitted to the ICU but all patients presenting to the hospital. This broad inclusion criteria improves generalizability of our findings. We found that elastic net regression was the most accurate algorithm for predicting in-hospital mortality in our datasets. The value of elastic net regression machine learning algorithms is that it is interpretable. We provide the variables and the coefficients for each model to ease future testing in diverse patient cohorts.

The present machine learning models show that a reliable prediction can be made for hospital mortality and organ failure in hospitalized patients with COVID-19. The AUC for our model had a performance in the external validation set comparable to or improved than alternative COVID-19 prediction models[12,29-31]. One benefit of our model is that it was developed and internally validated in a US population and externally validated in a population from China. This is in contrast to other prediction models developed in COVID-19 that are specific to patients admitted to one healthcare system or hospitalized in one country[12,13,28,29,32,33]. The ability to validate our model in a healthcare system outside the US shows the generalizability of the model and the reproducibility of our findings. Our findings also demonstrate the inherent similarities in the patient response to infection and the clinical variables that are associated with poor outcomes.

This study has several strengths, including its large sample size and a discovery and validation cohort. In addition, we developed models for not only mortality but also organ specific failure. Another strength is that the model predicted outcomes up to 2 weeks prior to the outcome occurring. This lead time is essential to help inform clinical care and provide a window when therapeutics can be tested to change eventual outcomes. Finally, all prediction models were developed using routinely collected data that is available in most electronic medical records. This allows the easy replication of our models to diverse patient cohorts. Since age is one of the strongest predictors of mortality in COVID-19, we specifically developed in-hospital mortality prediction models in the population of patients > 50 years of age. We found that clinical biomarkers, such as platelet count, blood urea nitrogen, white blood cell count and blood urea nitrogen, in combination continued to accurately predict in-hospital mortality.

There are also several limitations to this work. First, although developed and validated in an external dataset, it is possible that our findings may not generalize to other settings. Second, we restricted to clinical and laboratory variables collected within 24 hours of ICU admission. We restricted to these variables to develop prediction models that could be run on electronic health record data. Moreover, the variables used in the model are often not missing in the medical record and regularly collected.

## Conclusions

We developed prediction models with high discrimination for mortality, shock and RRT. The in-hospital mortality model performed well in the internal validation set and showed improved accuracy in the external validation set. Key variables that informed the in-hospital mortality prediction model included age, white blood cell count and platelet count. The mortality prediction model on average was able to identify future risk of mortality 2 weeks prior to the clinical outcome. All variables to develop the prediction models used clinical variables collected within the first day of hospital admission. These machine learning derived prediction models could be used to improve triage decisions, resource allocation, and support clinical trial enrichment in patients hospitalized with COVID-19.

## Supplementary Material

Supplement 1

## Figures and Tables

**Table 1A.** Features in the UW dataset stratified by survivors and non-survivors.

	Total (n=712)	Non-survivors (n=122)	Survivors (n=590)
Age, years	57 (44,69)	66 (54.25,75)	55 (41,66)
Female, n (%)	267 (38)	37 (30)	230 (39)
Male, n (%)	445 (62)	85 (70)	360 (61)
Maximum Serum Creatinine, mg/dL	0.97 (0.73,1.5)	1.16 (0.77,2.5)	0.95 (0.72,1.4)
Minimum Serum Creatinine, mg/dL	0.83 (0.64,1.19)	1.05 (0.67,1.82)	0.8 (0.63,1.11)
Maximum White Blood Cell Count, per mm^3^	8.11 (5.81,12.12)	9.85 (7.01,14.44)	7.87 (5.64,11.37)
Minimum White Blood Cell Count, per mm^3^	6.72 (4.8,9.89)	7.34 (5.28,11.17)	6.53 (4.63,9.63)
Maximum Glucose, mg/dL	138 (111,186.5)	154.5 (118,236)	135 (109,182)
Minimum Glucose, mg/dL	108 (92,133)	111.5 (95,138)	106.5 (91,132)
Maximum Serum Potassium, mmol/L	4.1 (3.8,4.6)	4.4 (4,4.8)	4.1 (3.8,4.6)
Minimum Serum Potassium, mmol/L	3.7 (3.4,4)	3.8 (3.5,4.2)	3.7 (3.4,4)
Maximum Platelet Count, 10^9^/L	223.5 (176,302)	190 (138.5,254.25)	228.5 (183.75,312)
Minimum Platelet Count, 10^9^/L	194 (148, 259)	155 (114, 234)	200 (155, 265)
Maximum Serum Sodium, mmol/L	137 (134,140)	137 (134,140.25)	137 (135,140)
Minimum Serum Sodium, mmol/L	135 (132,138)	134 (132,138)	135 (132,137)
Maximum Serum Chloride, mmol/L	103 (100,106)	103 (98.75,107.25)	103 (100,106)
Minimum Serum Chloride, mmol/L	100 (97,103)	99 (95,104)	100 (97,103)
Maximum Hematocrit, %	38 (33,42)	36 (32,41)	38 (34,43)
Minimum Hematocrit, %	35 (30,39)	34 (29,38)	35 (31,39)
Maximum Blood Nitrogen Urea, mg/dL	19.5 (13,33)	30 (17,54)	19 (13,31)
Minimum Blood Nitrogen Urea, mg/dL	16 (11,27)	23 (15,39.25)	15 (10,24)

All variables are median and interquartile range unless otherwise specified.

**Table 1B.** Features in the Tongji dataset stratified by survivors and non-survivors.

	Total (n=345)	Non-survivors (n=159)	Survivors (n=186)
Age, years	62 (46,70)	69 (63,77.5)	51 (37,62.75)
Female, n (%)	143 (41)	43 (27)	100 (54)
Male, n (%)	202 (59)	116 (73)	86 (46)
Maximum Serum Creatinine, mg/dL	0.86 (0.66, 1.1)	1 (0.79, 1.29)	0.72 (0.6, 0.97)
Minimum Serum Creatinine, mg/dL	0.86 (0.64, 1.1)	0.98 (0.76, 1.28)	0.72 (0.6, 0.97)
Maximum White Blood Cell Count, per mm^3^	7.2 (4.75, 12.89)	10.75 (7.08, 15.97)	5.38 (4.15, 7.59)
Minimum White Blood Cell Count, per mm^3^	5.7 (4.08, 9.09)	9.14 (6.07, 13.4)	4.61 (3.6, 5.8)
Maximum Glucose, mg/dL	125 (104, 164)	151 (119, 204)	109 (94, 138)
Minimum Glucose, mg/dL	124 (104, 163)	150 (118, 203)	109 (94, 138)
Maximum Serum Potassium, mmol/L	4.2 (3.9,4.6)	4.3 (3.9,4.8)	4.1 (3.8, 4.5)
Minimum Serum Potassium, mmol/L	4.2 (3.8,4.6)	4.3 (3.9,4.7)	4.1 (3.8, 4.5)
Maximum Platelet Count, 10^9^/L	179 (134,231)	149 (109,212)	201 (160,254)
Minimum Platelet Count, 10^9^/L	177 (134,231)	149 (107,206)	201 (160,254)
Maximum Serum Sodium, mmol/L	139 (136, 142)	139 (136, 144)	139 (136, 141)
Minimum Serum Sodium, mmol/L	139 (136,142)	139 (136,144)	139 (136, 141)
Maximum Serum Chloride, mmol/L	101 (98,104)	101 (97,106)	101 (99, 103)
Minimum Serum Chloride, mmol/L	101 (98,104)	101 (97,105)	101 (99, 103)
Maximum Hematocrit, %	37 (34, 41)	37 (34, 41)	37 (34, 40)
Minimum Hematocrit, %	37 (34, 40)	36 (33, 41)	37 (34, 40)
Maximum Blood Nitrogen Urea, mg/dL	15 (11, 25)	25 (16, 36)	11 (9, 15)
Minimum Blood Nitrogen Urea, mg/dL	15 (11, 25)	25 (16, 36)	11 (9, 15)

**Table 2A.** Model performance in the training, internal and external validation sets for in-hospital mortality.

Test Sets	Statistics	Lasso LR	Elastic net LR	XGBoost
Training	Sensitivity (95% CI)	0.12 (0.06,0.22)	0.12 (0.06,0.22)	0.99 (0.93,1.0)
	Specificity (95% CI)	0.99 (0.98,1.0)	0.99 (0.98,1.0)	1.0 (0.99,1.0)
	AUC (95% CI)	0.76 (0.71,0.81)	0.78 (0.73,0.83)	1.0 (1.0,1.0)
Internal validation	Sensitivity (95% CI)	0.05 (0.01,0.17)	0.10 (0.03,0.23)	0.37 (0.22,0.53)
	Specificity (95% CI)	0.98 (0.95,0.99)	0.98 (0.95,0.99)	0.89 (0.84,0.93)
	AUC (95% CI)	0.68 (0.59,0.77)	0.72 (0.64,0.81)	0.67 (0.59,0.76)
External validation	Sensitivity (95% CI)	0.11 (0.06,0.16)	0.22 (0.16,0.28)	0.50 (0.42,0.57)
	Specificity (95% CI)	0.99 (0.97,1.0)	0.97 (0.94,0.99)	0.93 (0.89,0.97)
	AUC (95% CI)	0.83 (0.78,0.87)	0.85 (0.81,0.89)	0.77 (0.72,0.82)

**Table 2B.** Model performance in the training, internal and external validation sets for in-hospital mortality for patients over 50.

Test Sets	Statistics	Lasso LR	Elastic net LR	XGBoost
Training	Sensitivity (95% CI)	0.06 (0.02,0.14)	0.25 (0.16,0.36)	0.99 (0.93,1.0)
	Specificity (95% CI)	0.98 (0.96,0.99)	0.95 (0.91,0.97)	1.0 (0.99,1.0)
	AUC (95% CI)	0.68 (0.62,0.75)	0.7 (0.64,0.77)	1.0 (1.0,1.0)
Internal validation	Sensitivity (95% CI)	0.05 (0,0.3)	0.15 (0.03,0.38)	0.45 (0.23,0.68)
	Specificity (95% CI)	1.0 (0.95,1.0)	0.97 (0.9,1.0)	0.91 (0.82,0.97)
	AUC (95% CI)	0.66 (0.54,0.79)	0.73 (0.61,0.84)	0.72 (0.6,0.85)
External validation	Sensitivity (95% CI)	0.05 (0.01,0.08)	0.27 (0.2,0.34)	0.43 (0.35,0.51)
	Specificity (95% CI)	0.97 (0.93,1.0)	0.95 (0.9,0.99)	0.89 (0.82,0.95)
	AUC (95% CI)	0.8 (0.75,0.86)	0.82 (0.76,0.87)	0.71 (0.66,0.77)

**Table 3A.** Negative and positive predictive values for the Elastic net LR model and outcome of in-hospital mortality.

	Performance Goal	Patients Above/Below Threshold	Sensitivity (95% CI)	Specificity (95% CI)	PPV (95% CI)	NPV (95% CI)
Training	Maximizing NPV	409/65	1 (0.96,1.0)	0.17 (0.13,0.21)	0.2 (0.16,0.24)	1 (0.94,1.0)
	Maximizing PPV	3/471	0.04 (0.01,0.1)	1.0 (0.99,1)	1.0 (0.29,1.0)	0.83 (0.8,0.87)
Internal validation	Maximizing NPV	165/73	0.93 (0.8,0.98)	0.36 (0.29,0.43)	0.23 (0.17,0.3)	0.96 (0.88,0.99)
	Maximizing PPV	1/237	0.02 (0,0.13)	1.0 (0.98,1.0)	1.0 (0.03,1.0)	0.83 (0.78,0.88)
External validation	Maximizing NPV	308/37	0.98 (0.95,1)	0.18 (0.13,0.25)	0.51 (0.45,0.56)	0.92 (0.78,0.98)
	Maximizing PPV	40/305	0.22 (0.16,0.29)	0.97 (0.94,0.99)	0.88 (0.73,0.96)	0.59 (0.54,0.65)

**Table 3B.** Negative and positive predictive values for the Elastic net LR model and outcome of in-hospital mortality for patients over 50.

	Performance Goal	Patients Above/Below Threshold	Sensitivity (95% CI)	Specificity (95% CI)	PPV (95% CI)	NPV (95% CI)
Training	Maximizing NPV	352/3	1 (0.95,1)	0.01 (0,0.03)	0.22 (0.18,0.27)	1 (0.29,1)
	Maximizing PPV	5/350	0.04 (0.01,0.11)	0.99 (0.97,1)	0.6 (0.15,0.95)	0.78 (0.74,0.82)
Internal validation	Maximizing NPV	76/13	1 (0.83,1)	0.19 (0.1,0.3)	0.26 (0.17,0.38)	1 (0.75,1)
	Maximizing PPV	1/88	0.05 (0,0.25)	1 (0.95,1)	1 (0.03,1)	0.78 (0.68,0.86)
External validation	Maximizing NPV	192/55	0.94 (0.89,0.97)	0.47 (0.37,0.58)	0.73 (0.67,0.8)	0.84 (0.71,0.92)
	Maximizing PPV	56/191	0.33 (0.26,0.41)	0.94 (0.87,0.98)	0.89 (0.78,0.96)	0.48 (0.4,0.55)

**Table 4.** Model performance by 10-fold cross validation for ICU transfer, shock, and RRT. The sensitivity and specificity were calculated at the cut-off value of 0.5.

Outcome	Statistics	Lasso LR	Elastic net LR	XGBoost
ICU transfer	Sensitivity (95% CI)	0 (0,0.08)	0.02 (0,0.12)	0.02 (0,0.12)
	Specificity (95% CI)	1 (0.99,1)	1 (0.99,1)	0.92 (0.89,0.95)
	AUC (95% CI)	0.6 (0.52,0.68)	0.58 (0.5,0.66)	0.51 (0.36,0.65)
Shock	Sensitivity (95% CI)	0.03 (0,0.1)	0.03 (0,0.1)	0.45 (0.33,0.57)
	Specificity (95% CI)	0.99 (0.98,1)	0.99 (0.98,1)	0.91 (0.89,0.94)
	AUC (95% CI)	0.75 (0.68,0.83)	0.76 (0.69,0.82)	0.7 (0.58,0.82)
RRT	Sensitivity (95% CI)	0.25 (0.1,0.47)	0.25 (0.1,0.47)	0.58 (0.37,0.78)
	Specificity (95% CI)	0.99 (0.98,1)	0.99 (0.98,1)	0.98 (0.97,0.99)
	AUC (95% CI)	0.88 (0.79,0.98)	0.88 (0.78,0.98)	0.78 (0.6,0.95)
