# Supplementary material for: Machine Learning-based Derivation and External Validation of a Tool to Predict Death and Development of Organ Failure in Hospitalized Patients with COVID-19
Source: Res Sq. 2021 Nov 16:rs.3.rs-1009310. Preprint. [Version 1] doi: 10.21203/rs.3.rs-1009310/v1 (PMC8609901; doi:10.21203/rs.3.rs-1009310/v1)
Supplement: Supplement 1 [file caaff6852b5432b2d8fa753e.docx]

**ONLINE SUPPLEMENTARY MATERIALS**

**Title:** Machine learning-based derivation and external validation of a tool to predict death and development of organ failure in hospitalized patients with COVID-19

**Authors**: Xu, Y.; Trivedi, A.; Becker N.; et al

**Table of Contents**

**Figure S1. Calibration plots for prediction models**.

**Figure S2. Variable importance plots.**

**Table S1. Elastic net LR model for in-hospital mortality prediction**

**Table S2. Lasso LR model for ICU transfer prediction**

**Table S3. Elastic net LR model for shock prediction**

**Table S4. Lasso LR model for dialysis prediction**

**List of Variables**

**Figure S1. Calibration plots for prediction models**. A. 28-days mortality in the internal validation. B. 28-days mortality in the external validation. C. 28-day ICU transfer in the internal validation D. 28-day shock in the internal validation. 28-day receipt of RRT in the internal validation.


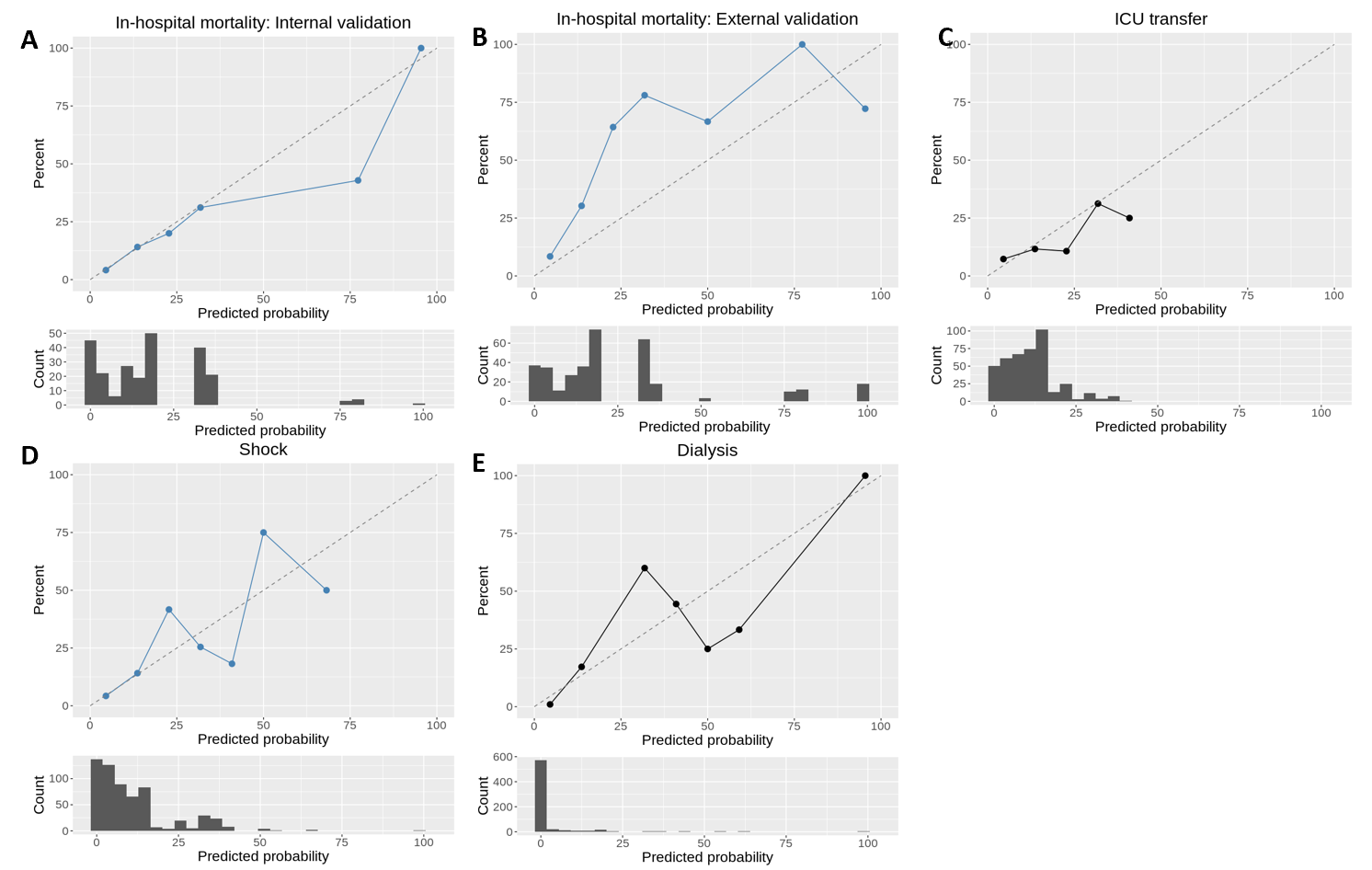


**Figure S2. Variable importance plots.** (B) 28-day shock (C) and 28-day dialysis (D) prediction models by SHAP


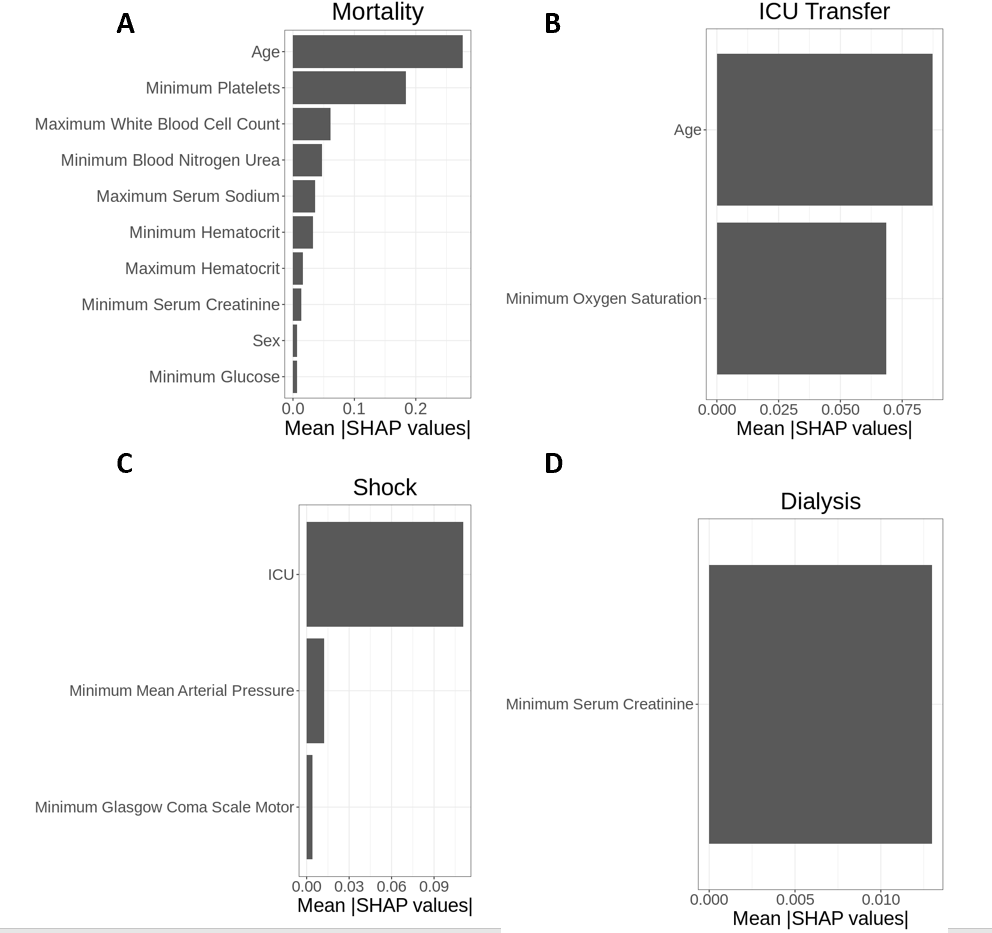

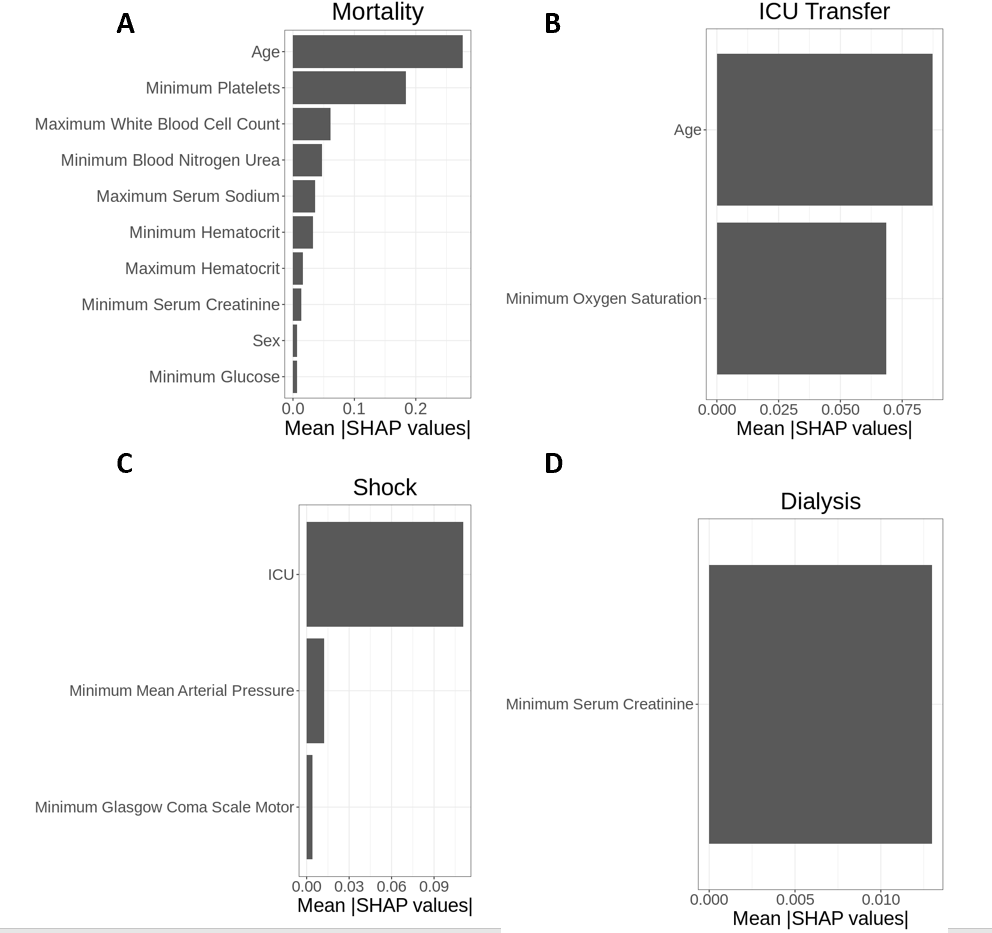


**Table S1. Elastic net LR model for in-hospital mortality prediction**

| Variable name | Coefficient |
| --- | --- |
| Intercept | -2.02165 |
| Age | 0.019197 |
| Sex | 0.013532 |
| Minimum Serum Creatinine | 0.016108 |
| Maximum White Blood Cell Count | 0.01365 |
| Minimum Glucose | 0.000193 |
| Minimum Platelets | -0.00259 |
| Maximum Serum Sodium | 0.010451 |
| Maximum Hematocrit | -0.00328 |
| Minimum Hematocrit | -0.00665 |
| Minimum Blood Nitrogen Urea | 0.004135 |

**Table S2. Lasso LR model for ICU transfer prediction**

| Variable name | Coefficient |
| --- | --- |
| Intercept | 2.249323 |
| Age | 0.005735 |
| Minimum oxygen saturation | -0.02782 |

**Table S3. Elastic net LR model for shock prediction**

| Variable name | Coefficient |
| --- | --- |
| Intercept | -0.00931 |
| Minimum Mean Arterial Pressure | -0.00121 |
| Minimum Glasgow Coma Scale Motor | -0.00588 |
| ICU | 0.254973 |

**Table S4. Lasso LR model for dialysis prediction**

| Variable name | Coefficient |
| --- | --- |
| Intercept | -0.05697268 |
| Minimum Serum Creatinine | 0.02231206 |

**List of Variables**

**The full list of variables for mortality prediction using the overlapping features:**

Age, Maximum Blood Nitrogen Urea, Maximum Glucose, Maximum Hematocrit, Maximum Platelets, Maximum Serum Chloride, Maximum Serum Creatinine, Maximum Serum Potassium, Maximum Serum Sodium, Maximum White Blood Cell Count, Minimum Blood Nitrogen Urea, Minimum Glucose, Minimum Hematocrit, Minimum Platelets, Minimum Serum Chloride, Minimum Serum Creatinine, Minimum Serum Potassium, Minimum Serum Sodium, Minimum White Blood Cell Count, Sex.

**The full list of variables for ICU transfer prediction**:

Age, Dialysis, Ethnicity, Height, Maximum Anion Gap, Maximum Blood Nitrogen Urea, Maximum Glasgow Coma Scale Motor, Maximum Glasgow Coma Scale Total, Maximum Glasgow Coma Scale Verbal, Maximum Glasgow Coma Scale Visual, Maximum Glucose, Maximum Hematocrit, Maximum Mean Arterial Pressure, Maximum Platelets, Maximum Pulse, Maximum Respiratory Rate, Maximum Serum Bicarb, Maximum Serum Chloride, Maximum Serum Creatinine, Maximum Serum Potassium, Maximum Serum Sodium, Maximum Systolic Blood Pressure, Maximum Temperature, Maximum White Blood Cell Count, Minimum Anion Gap, Minimum Blood Nitrogen Urea, Minimum Glasgow Coma Scale Motor, Minimum Glasgow Coma Scale Total, Minimum Glasgow Coma Scale Verbal, Minimum Glasgow Coma Scale Visual, Minimum Glucose, Minimum Hematocrit, Minimum Mean Arterial Pressure, Minimum oxygen saturation, Minimum Platelets, Minimum Pulse, Minimum Respiratory Rate, Minimum Serum Bicarb, Minimum Serum Chloride, Minimum Serum Creatinine, Minimum Serum Potassium, Minimum Serum Sodium, Minimum Systolic Blood Pressure, Minimum Temperature, Minimum White Blood Cell Count, Race, Sex.

**The full list of variables for shock prediction**:

Age, Blood Product - FFP Day 1, CAM ICU, Daily Net Fluid Balance Day 1, Dialysis, Ethnicity, Height, ICU, Intubatated, Maximum Anion Gap, Maximum Blood Nitrogen Urea, Maximum Glasgow Coma Scale Motor, Maximum Glasgow Coma Scale Total, Maximum Glasgow Coma Scale Verbal, Maximum Glasgow Coma Scale Visual, Maximum Glucose, Maximum Hematocrit, Maximum Mean Arterial Pressure, Maximum Platelets, Maximum Pulse, Maximum Respiratory Rate, Maximum Serum Bicarb, Maximum Serum Chloride, Maximum Serum Creatinine, Maximum Serum Potassium, Maximum Serum Sodium, Maximum Temperature, Maximum White Blood Cell Count, Minimum Anion Gap, Minimum Blood Nitrogen Urea, Minimum Glasgow Coma Scale Motor, Minimum Glasgow Coma Scale Total, Minimum Glasgow Coma Scale Verbal, Minimum Glasgow Coma Scale Visual, Minimum Glucose, Minimum Hematocrit, Minimum Mean Arterial Pressure, Minimum oxygen saturation, Minimum Platelets, Minimum Pulse, Minimum Respiratory Rate, Minimum Serum Bicarb, Minimum Serum Chloride, Minimum Serum Creatinine, Minimum Serum Potassium, Minimum Serum Sodium, Minimum Temperature, Minimum White Blood Cell Count, Race, Sex.

**The full list of variables for dialysis prediction:**

Age, Blood Product - FFP Day 0, Blood Product - FFP Day 1, CAM ICU, Daily Ins Day 1, Daily Net Fluid Balance Day 1, Ethnicity, Height, ICU, Intubatated, Maximum Anion Gap, Maximum Blood Nitrogen Urea, Maximum Glasgow Coma Scale Motor, Maximum Glasgow Coma Scale Total, Maximum Glasgow Coma Scale Verbal, Maximum Glasgow Coma Scale Visual, Maximum Glucose, Maximum Hematocrit, Maximum Mean Arterial Pressure, Maximum Platelets, Maximum Pulse, Maximum Respiratory Rate, Maximum Serum Bicarb, Maximum Serum Chloride, Maximum Serum Creatinine, Maximum Serum Potassium, Maximum Serum Sodium, Maximum Systolic Blood Pressure, Maximum Temperature, Maximum White Blood Cell Count, Minimum Anion Gap, Minimum Blood Nitrogen Urea, Minimum Glasgow Coma Scale Motor, Minimum Glasgow Coma Scale Total, Minimum Glasgow Coma Scale Verbal, Minimum Glasgow Coma Scale Visual, Minimum Glucose, Minimum Hematocrit, Minimum Mean Arterial Pressure, Minimum oxygen saturation, Minimum Platelets, Minimum Pulse, Minimum Respiratory Rate, Minimum Serum Bicarb, Minimum Serum Chloride, Minimum Serum Creatinine, Minimum Serum Potassium, Minimum Serum Sodium, Minimum Systolic Blood Pressure, Minimum Temperature, Minimum White Blood Cell Count, Race, Sex, Shock.
